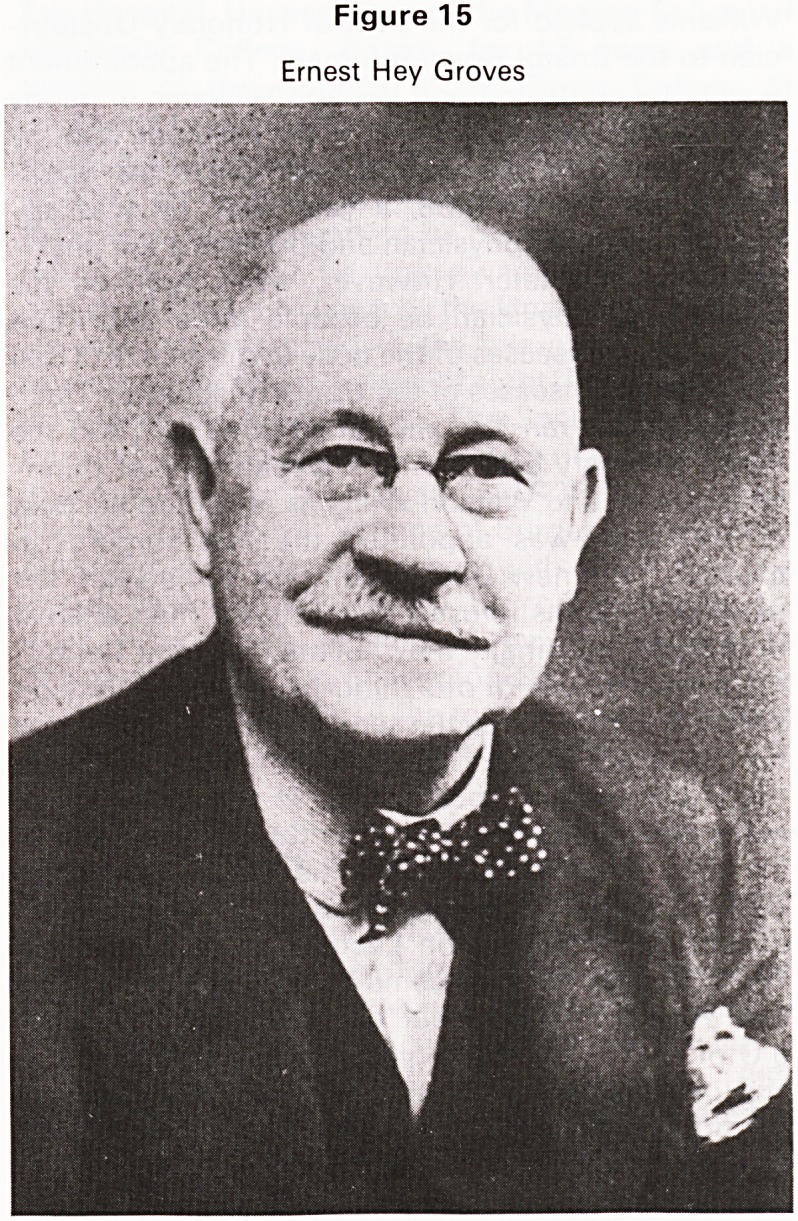# Some Famous Bristol Doctors

**Published:** 1983

**Authors:** C. Bruce Perry

**Affiliations:** Emeritus Professor of Medicine, University of Bristol


### Some Famous Bristol Doctors

**C. Bruce Perry, M.D., F.R.C.P.**
Emeritus Professor of Medicine, University of Bristol.

### THOMAS DOVER

In 1696 an Act of Parliament established the Bristol Corporation of the Poor. This brought together the Poor Law arrangements of all the parishes in the City. It was hoped that the inmates by their work would produce goods which would have a ready sale and thus defray the costs. For this purpose the Aldworth mansion was purchased from a group of merchants, one of whom was Edward Colston. This building after ceasing to be a private residence had first housed the Bristol Mint and more recently had been a sugar refinery. The 'union' opened in 1698 and *Dr. Thomas Dover* offered his services as a physician free of charge for two years. He insisted on the inmates having a diet better than that of the average employed labourer. The building situated near St. Peter's Church soon became known as St. Peter's Hospital (Figure 1). At first all went well, but the sale of products was not a success and conditions deteriorated, and before long the aged, infirm and lunatics preponderated. In 1786 a report by Sir Thomas Eden noted, apart from overcrowding, filth, bugs and vermin. The building survived as the headquarters of the Guardians of the Poor until destroyed by enemy action in 1940.

Thomas Dover was born in 1662 near Moreton-in-the-Marsh. He graduated B.A. from Magdalen Hall, Oxford, in 1684. After being a clinical assistant to Sydenham for two years he was admitted to Caius College, Cambridge, and graduated B.M. in 1687. He set up in Bristol in 1691. After his two years service to St. Peter's Hospital he apparently decided that trading with the West Indies, presumably with slaves, was more profitable than treating patients in Bristol. Between 1701 and 1707 he made several voyages to the Caribbean as a part owner of the ship and as a ship's doctor, and was often referred to as Captain Dover. In 1708 he set sail from Bristol with Woodes Rogers's privateering expedition to circumnavigate the world as one of the owners in the Duke and Duchess. This journey took nearly four years, the ships returning to the Thames in 1711. Throughout the health of the ships' crews was, for that time, surprisingly good, probably because of Dover's influence. He was not actually the ships' surgeon, but as he had subscribed the second largest sum to finance the expedition, he sailed to represent the owners and was entitled to preside over the council of the captains and navigators and to have two votes.

**Figure 1**

St. Peter's Hospital

The ships carried two official surgeons, a surgeon's mate, an assistant surgeon, and an apothecary. In his capacity as representing the owners Dover soon showed his cantankerous nature and fell out with Woodes Rogers so badly that they sailed in different ships. Finally he insisted on sailing as commander of one of the Spanish ships they had captured. After much argument this was agreed but Dover was forbidden to interfere in any way with the navigation of the ship. During the journey the expedition called at the Island of Juan Fernandez and there rescued Alexander Selkirk, who had been marooned alone for four years and four months. On the return Selkirk's story formed the basis of Daniel Defoe's 'Robinson Crusoe'. Whether Defoe ever actually met Selkirk or not is uncertain. This expedition, too, saw the capture and sacking of the Spanish town Guayaquel in which Dover played a prominent part. He became enthusiastic over South Sea trade, mortgaged his property on the Cotswolds, and invested all his money in the South Sea Company and in 1714 was appointed President of the Company at Buenos Aires. This appointment was terminated after two years, possibly because he had been engaging in some private trading. When the South Sea Bubble burst he was ruined. He struggled, not very successfully, to collect a practice in Bristol and in 1720 was admitted a licentiate of the Royal College of Physicians and moved to London. However, shortly afterwards a Dr. Wagstaffe complained to the College about his professional behaviour, but after an enquiry Dover was only 'admonished'. This may have been because he was a friend of Sir Hans Sloane, the President. Incensed by this he wrote a book 'The Ancient Physician's Legacy to his Country'. This was a 'do it yourself' textbook of medicine which ran into six editions, the last in 1742. In this he attacked the College and the practices of the Fellows, who 'like moles work underground lest their practices should be discovered to the Populace'. As a doctor he appears to have been fairly sound. In the introduction to the Legacy he wrote: 'It is essentially necessary in the Cure of Diseases to be thoroughly acquainted with the Nature of them. Without this knowledge no good is to be done.' Nowadays we remember him as the originator of Dover's Powder – pulv.ipecac.et opii. But in his own time he was notorious for his use of mercury as a panacea for nearly all ills and was often known as the 'Quick silver Doctor'. This was much criticised and one of his critics recorded the following: 'I have heard a pleasant story of a mercurial lady, who in dancing at a Public Assembly, happened to let go some particles of the quick silver she had taken in the morning; which shining on the floor in the midst of so great an illumination like so many brilliants, there were several stooping down to take them up . . .' His therapeutics were certainly vigorous at times but he attributes to Sydenham 12 bottles of Small Beer acidulated with Spirits of Vitriol every 24 hours for a case of small-pox. Dover died in 1742, widowed, homeless and virtually bankrupt, at the home of a friend at Stanway Hall on the Cotswolds and is buried in the Tracey vault in the church.

### JOHN BONYTHON

Shortly after Dover left Bristol, a Cornishman named John Bonython (Figure 2) arrived to practice in the City. Born in 1695 he was educated at Eton and King's College, Cambridge, where he had been elected a Fellow. He practised in Park Row, but it is said that being in easy circumstances he was not

**Figure 2**

John Bonython much concerned with private practice. He was, however, much concerned with the establishment of the Bristol Infirmary and has been called the Father of the Charity. There is little doubt that if it had not been for the work and enthusiasm of John Bonython and of John Ellbridge, the Controller of H.M. Customs, the Infirmary would not have been opened in 1737. In December 1736 Bonython wrote to a friend, John Orlebar 'For this half year I have been working hard at a scheme which if I can bring it to bear will make a vary great alteration in my way of living. It is to set up in this rich and populous city an Infirmary for sick and wounded by an annual subscription as is done at St. James's Westminster and Hyde Park Corner and lately at Winchester.' When the Infirmary was founded he was appointed the first Physician. He seems to have become rather autocratic, as after a while the rest of the staff were concerned because he insisted on seeing all patients to be admitted, including those recommended by the surgeons. He died in 1761. A board to his memory was erected, the inscription on which reads as follows: 'The growth and improvement of this Charity have been greatly owing to his care and conduct. He was zealous and indefatigable in promoting its success and watchful in directing every occurrence to its advantage. He always had in his view the Plan and Design of this benevolent institution, viz.: the care and relief of the laborious Sick and distressed Poor, whose good he had at heart: no calamitous objects presented themselves but he participated in their grief and with a Christian concern and Brotherly tenderness felt their pains. This was the principle that made him active in affording his most generous and kind assistance: and we presume tho' now alas he is dead, yet he will live for ages in the grateful acknowledgements of thousands and in the good esteem of all.'

### EDWARD LONG FOX SENIOR

The most distinguished Bristol physician at the end of the eighteenth and beginning of the nineteenth centuries was Edward Long Fox Senior (Figure 3). Another Cornishman, he was born at Falmouth in 1761, where his father was in practice as a surgeon and apothecary. At the age of 18 he was apprenticed

**Figure 3**

Edward Long Fox Senior to his father, but the same year he entered the University of Edinburgh. However, in 1781 he returned to Falmouth as an assistant to his father for two years. After going back to Edinburgh, he graduated M.D. in 1784, the title of his graduation thesis being 'De voce humana'. He moved to Bristol in 1786 and set up in practice in Castle Green. He was elected physician to the Infirmary in April of that year. The election, as was usual at that time, was closely fought and quite a public affair. Long Fox, as a Quaker, was supported by Messrs. Harford, Battersly and Butler, all Quakers. His main opponent, Dr. Cave, had the strong support of Messrs. Fry and Cave, distillers and wine merchants. The contest thus became known as The Distillers versus The Quakers. In a subsequent election Long Fox voted at the door as a member of the staff, as he was entitled to do, but later voted again as a subscriber. When challenged and accused of voting twice, he replied that 'he really didn't recollect it, but he believed he had'. Munro Smith, the Infirmary historian, excuses this as being 'of course, mere absence of mind'. As a Quaker he was a strong supporter of the anti-slavery movement. His father, in addition to his medical practice, was a member of a firm owning ships. At the outbreak of the war with France, these ships were fitted with guns as privateers and made some valuable captures. His share of these amounted to £22,000, but his Quaker principles made him strongly disapprove. He made enquiries and discovered who in France had suffered the loss and at the end of the war Edward was sent to France to refund the money. A Bristol paper, learning of this, published the following doggerel:

> A doctor well skill'd in the medical art
> 'Mongst others for France was resolved to depart
> And leave his domestic concerns –
> But what will become of his patients the while?
> 'O fear not' a neighbour replied with a smile!
> 'They will *live* – till the doctor returns'.

In 1793 he moved to Queen Square and became more and more interested in the humane treatment of the insane as opposed to the barbarities in use at Bedlam. Although a pioneer in this, he may have been influenced to some extent by the work going on at this time at the Retreat in York. In 1784 he succeeded Dr. Henderson in charge of a private asylum at Downend, but in 1804 he built Brislington House which was opened in 1806. Here chains and intimidation were abolished and games, drives, occupational pursuits and regular church services took their place. Some patients were provided with their own houses. At one time he was severely criticised for studying Mesmer's Animal Magnetism (Hypnotism) and for trying it on some patients. His reply was that 'The experimental enquiry was begun from most disinterested motives, but that being unable to ascertain that any such power as animal magnetism existed he had laid it altogether aside'. As an authority he was called to Windsor in consultation on George III during one of the monarch's relapses. In addition to his interest in lunacy, he speculated on the 'animalcular' origin of disease and in 1831 published 'Surmises respecting the Cause and Nature of Cholera'. Retiring from the Infirmary in 1816 he devoted himself to Brislington House, but in addition he bought Knightstone at Weston-super-Mare, where he built salt water baths 'chiefly for the use of Infirmary patients'. However, it is not clear how they got there. He was married twice and had twenty-two children, four of whom became doctors and one, Henry Hawes Fox, succeeded his father as physician to the Infirmary from 1816 to 1829. He died in 1835, aged seventy-four.

### THOMAS BEDDOES

In 1793 Dr. Thomas Beddoes (Figure 4) retired from his appointment as Reader in Chemistry at Oxford as a result of his unpopularity there which stemmed from his pamphlets expressing sympathy with, and approval of, the French Revolutionaries. He came to Bristol and lived at 3 Rodney Place. Long interested in the chemistry of gases, he conceived the idea that their inhalation would be of value in the treatment of disease. His first venture was to establish a laboratory in Hope Square for the preparation of various gases or facititious airs as they were called. He poured scorn, correctly, on the claims of the Hotwells for the treatment of consumption, which was already falling into disrepute. Fairly rapidly he developed quite a practice and became very friendly with the literary circle in Bristol, which included Coleridge, Southey and Wordsworth, and he married Anna Maria, Maria Edgeworth's elder sister. In 1799 he established at 6 Dowry Square a small hospital, designed to make use of the 'factitious airs', known as the Pneumatic Medical Institute. Needing someone to supervise the preparation of the gases he appointed the young Humphry Davy, then aged 19, who had been recommended to him by friends in Cornwall. In the Institute Davy carried out many experiments on the effects of the 'laughing gas' (Nitrous oxide), using himself and others, including Coleridge, Southey and 'some young lady friends'. As a result of these experiments Davy anticipated its use as an anaesthetic when in 1800 he wrote that it might prove of use during surgical operations, since it appeared to abolish physical pain. After Davy moved to London in 1803 Beddoes gradually gave up the use of gases and the Institute was moved to Broad Quay, where it lingered until about 1809. Beddoes was never appointed to the staff of the Infirmary, but in 1798, following the habit which led to his leaving Oxford, he published a pamphlet entitled 'A suggestion towards an essential improvement in the Bristol Infirmary'. In this he recommended that two physicians and four surgeons should retire annually. It can be understood that this did not commend him to the Infirmary staff. Another of his pamphlets was an early attempt at preventive medicine entitled 'A Guide for Self Preservation and Parental Affection'. In this he stressed the importance of good diet, cleanliness and fresh air. In 1797 with F. C. Bowles, an Infirmary surgeon, he advertised a course of lectures on Anatomy to be given at the Red Lodge. The first lecture was to be by Beddoes. At the time appointed on November 17th the company were assembled but no lecturer. After some time Bowles 'ran to Clifton' and came back very out of breath with the lecture which Beddoes had hardly finished and which Bowles had to read, but 'found it difficult to decipher.' His use of gases led to much scoffing by the orthodox practitioners who regarded him as a quack. Rhymes such as the following were circulated about his activities:

**Figure 4**

Thomas Beddoes

'Nor boast thy airs cosmetic powers alone:
Disease and vanquished time their virtues own.

Pneumatic art unfixes cancer's claw
And shields the victim doomed to Phthisis maw,
See palsy dance, his hollow macies fill,
And asthma pace without a puff up hill.'

It is alleged that he recommended the treatment of consumption by the inhalation of cow's breath and that for this purpose a cow should be installed in the patient's bedroom with her muzzle inserted between the bed curtains and left there all night. This led a critic to write 'Do you want your wards turned into cow houses and your apothecaries' shop into a manufactory of gases?'

Beddoes died at the relatively early age of 48 in 1808.

### JAMES COWLES PRICHARD

James Cowles Prichard (Figure 5) was appointed physician to St. Peter's Hospital in 1811. Born in 1786 at Ross-on-Wye of a Quaker family he had been 'steeped in philosophic liberalism'. He had been apprenticed to Dr. Pole who practised in Bristol and then went to St. Thomas' Hospital where he studied anatomy and finally completed his clinical studies in Edinburgh where he graduated M.D. in 1809 with a thesis entitled 'De Humani Generis Varietate'. After Edinburgh he spent a few terms at Oxford, at first at St. John's 'where not finding the society congenial' he entered as a Gentleman Commoner at Trinity. His period at St. Peter's Hospital gave him the opportunity of making an intensive study of lunacy on which he became a great authority, contributing many articles on it and formulating the concept of 'moral insanity'. In 1814 he was appointed Physician to the Infirmary. Henry Alford, who was a student in 1822, recorded that although Prichard was the junior physician at that time he was far in advance of the others in culture, in general and professional knowledge and in literary reputation. He belonged to the depleting school of therapeutics and one of his patients wrote:

Dr. Prichard do appear
With his attendance and his care
He fills his patients full of sorrow
You must be bled today and cupped tomorrow.

He also had a great belief in counter irritation. His treatment for diseases of the brain like hemiplegia was by an incision of the scalp along the sagittal suture kept open by the insertion of peas. This was long known as Prichard's incision. Nevertheless it is recorded that as a physician he was distinguished 'by the earnestness with which he devoted himself to his duties and by his kind and considerate conduct towards his patients, and further that he kept detailed notes of his Infirmary patients in short, terse latin sentences'. But his work as an anthropologist is what really made him famous and for which he is mainly remembered. As a side line to this he wrote on Egyptian Mythology in 1813. His original thesis was gradually expanded into two volumes and ran into three editions. In this he concluded that all human races are of one species and one family. He speculated that man originally had a black skin and that the white races were produced 'under the influence of civilisation'. In view of the discovery of very early human remains in Africa by Leakey and others this may well be true. He was also far ahead of his time in concluding firmly that acquired pecularariarities are never transmitted to the offspring. In 1831 he was active in promoting the foundation of the short-lived Bristol College. Many honours came his way: M.D. Oxford by Diploma in 1835, Fellow of the Royal Society, Corresponding member of the National Institute of France and of the French Academy of Medicine. During most of his time in Bristol he lived

**Figure 5**

James Cowles Prichard

JAMES COWLES PRICHARD.

at the Red Lodge, but in 1845 on appointment as a Commissioner in Lunacy he moved to London, where he died three years later.

### JOHN ADDINGTON SYMONDS

By 1830 it was clear that the Infirmary could not deal with all the patients seeking its help and a group of benevolent persons, mostly Quakers, formed a committee with the object of doing something to rectify things. The result was the foundation of the General Hospital which, despite the difficulties occasioned by the Bristol riots and widespread cholera epidemic, opened its doors to patients in November 1832. The first physician appointed to the new hospital was John Addington Symonds (Figure 6). He was born in 1807 in Oxford where his father was a medical practitioner. At Magdalen College School he distinguished himself in classical studies. Moving to Edinburgh he graduated M.D. in 1828 and for three years acted as assistant in his father's practice. In 1831 he came to Bristol and settled in Berkeley Square. Following his appointment to the General Hospital he became the first Lecturer in Forensic Medicine in the young medical school and later lectured also on the Practice of Medicine. Amongst other medical papers he wrote on Death by Chloroform and on the Criminal Responsibility of Lunatics. In the latter he strongly supported Prichard's views on Moral Insanity. His practice flourished and in 1843 he resigned from the Hospital due to pressure of private work and moved to Clifton Hill House in 1851. He enjoyed travel and made frequent excursions to the Continent. However, these can hardly have been restful. For instance, during three weeks one summer he visited Cologne, Berlin, Dresden, Switzerland, Prague, Vienna, Salzburg, Munich and the Rhine, 'omitting no matter of importance'. He was elected F.R.C.P. in 1857 and the following year gave the Goulstonian Lectures on 'Headache' which, according to a medical authority, he treated in a most exhaustive manner. He had five children, four of whom survived. One daughter married Sir Edward Strachey, Bt., of Sutton Court and was the mother of St. Loe Strachey of *Spectator* fame. His son, John Addington Symonds junior, was the famous Victorian author and critic whose magnum opus was a seven-volume work on the Renaissance in Italy. After his father's death he published a collection of his non-medical writings and poems under the title 'Miscellanies'. These included an essay on Beauty, which was discussed under six headings: Sensational Beauty, Intellectual Beauty, Moral Beauty, Emotional Beauty, Ideal Beauty and Uses of Beauty. Other essays were on Knowledge, on Apparitions and Sleep and Dreams. One of the poems, inspired by seeing Nicholas Poussin's painting "Et ego in Arcadia vixi", ran as follows:

**Figure 6**

John Addington Symonds

Ah happy youth! ah happy maid!
Take present pleasure while ye may;
Laugh, dance and sing in sunny glade;
Your limbs are light, your hearts are gay;
Ye little think there comes a day
('Twill come to you, it came to me)
When love and life shall pass away –
I too once dwelt in Arcady.

Addington Symonds died in 1871 and the obituary in the *British Medical Journal* described him as: 'cautious in diagnosis, vigorous in treatment'. With no rival in the West of England as a consultant physican 'his warm and generous feelings, directed and controlled by sound judgement, diffused a steady glow of beneficience around him'.

### WILLIAM BUDD

The Father of Epidemiology, William Budd (Figure 7) was born in 1811 at North Tawton where his

**Figure 7**

William Budd father was in practice. In 1838 he graduated M.D. with a thesis on Rheumatic Fever which was awarded the Gold Medal. After a few years as assistant to his father, where in the country practice he started to study the mode of spread of typhoid which was eventually the subject of his magnum opus. He moved to Bristol and was appointed physician to St. Peter's Hospital in 1842. Following a common sequence he became Physician to the Infirmary five year's later. Budd has been described as the first scientific physician on the staff of that institution. His enthusiasm and interest in his patients was such that when he walked down to the Infirmary as soon as he saw it 'like a boy within sight of his bathing place or cricket field, he could hardly restrain himself from setting off to run, in his anxiety to see how his patients were getting on'. He believed in giving the patients a good diet and it was reported to the committee investigating expenses that Dr. Budd ordered a very much larger proportion of 'Extraordinaries' (fowl, fish, eggs, broth, beef tea, wines and spirits) than any of his colleagues. One great service to the Infirmary was his activity in promoting the building of a pathological museum. As a result of the pressure of a very large practice he resigned from the Infirmary staff in 1862. When the British Medical Association held its annual meeting in Bristol in 1863 he gave an address on 'The Laws of Contagious Epidemics'. In this he maintained that many contagious diseases are due to minute living organisms. This was developed in his great book 'Typhoid Fever, its Nature, Mode of Spreading and Prevention', published in 1873. The case, he wrote, may be likened to that of a poppy or many another plant. Poppies, like contagious fevers, propagate themselves. When the seed capsule is ripe it drops off, but the capsule itself has to be broken up, often travelling long distances the while – before the numberless seeds it encloses are cast upon the soil to spring up as fresh poppies. And so in a measure with the fever seed also''. He showed how typhoid was spread by water and sewage. In addition to this his book is noteworthy for the splendid illustrations of the pathology of typhoid fever which have probably never been bettered. He was never in a hurry to rush into print with his ideas. In 1866 he wrote to a friend, Dr. George Paget of Cambridge, enclosing a sealed packet containing a memorandum on the Infectiousness of Phthisis. He requested that the packet should not be opened except upon his own request. Acting on instructions Paget sent the memorandum to the Lancet in 1867. In it Budd had written: 'The idea first came into my mind unbidden, so to speak, while I was walking on the Observatory Hill at Clifton in the second week of August 1856 . . . The long interval which has occurred between the summer of 1856 and the present date has been occupied in collecting data bearing on the various questions raised by this new theory . . . During the whole of this long time the subject has scarcely ever been absent from my mind.' In 1870 he was elected to the Fellowship of the Royal Society. He retired to Clevedon and died there in 1880. In the Dictionary of National Biography it is stated of William Budd that 'No physician in England, who during his lifetime showed anything like his penetration in the interpretation of zymotic diseases.' His younger brother Francis Nonus Budd was the first Chairman of Council of University College, Bristol, serving from 1876 to 1882.

### JOHN BEDDOE

Perhaps an even more distinguished Physical Anthropologist than Cowles Prichard was Dr. John Beddoe (Figure 8). Born at Bewdley in Worcestershire in 1826 he was articled at the age of 20 to a firm of solicitors and by this time he had already started making records of the hair colour, eye colour and complexion of those he met. He was a student at University College, London, from 1847 to 1852 and

**Figure 8**

John Beddoe during the vacations he went to Orkney and Shetland studying the physical characteristics of the inhabitants. Moving to Edinburgh he qualified in 1853 and went as a doctor to the Crimea. Returning from the war he settled in Clifton in 1857 and was elected Physician to the Royal Infirmary in 1862. It is stated that he was the first member of the honorary staff not to wear a hat in the wards. By this time he was already well known for his anthropological studies. He was elected to the Fellowship of the Royal Society in 1873 and in the same year became a Fellow of the Royal College of Physicians. One of the founders of the Bristol and Gloucestershire Archaeological Society, he served as President in 1890. His great book 'Races of Britain' was published in 1885, but he did not confine his observations to the inhabitants of Britain. Whenever opportunity offered he travelled the continent noting the characteristics of the inhabitants of the different countries. His ideas on medical education were clearly sound and in the address at the Annual Prize Distribution of the Bristol Medical School he said: 'Whatever the number of years allotted for medical education it is of great importance that the last of them should be occupied in honest and untrammelled efforts to acquire, in hospitals or dispensaries, a practical knowledge of medical work'. In the same address he said: 'In our own day, undoubtedly, the brilliant triumphs of surgery appeal more forcibly to the uneducated critic, if not to the educated one, than the more obscure and doubtful victories of medicine.' When the British Association for the Advancement of Science met in Bristol in 1875 he was invited to be President of the Anthropological section. However he declined this honour, saying 'I could not afford to let my scientific reputation injure my medical practice.' In 1904 he gave the first Long Fox Memorial Lecture on 'The Ideal Physician', and six years later his autobiography 'Memories of 80 years' was published. The University of Bristol appointed him Honorary Professor of Anthropology in 1910. He died in 1911 aged 85. On the occasion of the meeting of the British Association in Bristol in 1930 Sir Arthur Keith delivered a memorial lecture on Beddoe. In this he said: 'We owe more to him than to any other antropologist of the Victorian epoch.' He went on to urge the University to found a Chair of Anthropology in his memory. This, alas, has not yet materialised.

### WILLIAM GILBERT GRACE

In 1868 a young man already famous in a different capacity entered the Bristol Medical School. William Gilbert Grace (Figure 9) was born in 1848 at Downend into a family where there was great enthusiasm for cricket. Presumably as a result of his cricket he took eleven years to qualify after studying not only in Bristol but also at St. Bartholomews and the Westminster. Nevertheless he set up in practice in Stapleton Road where he was medical officer to Barton Regis Union and Public Vaccinator. Later he moved to Clifton, where he did quite a lot of insurance work, 'particularly in the winter months'. At the age of 17 he played for the Gentleman v. the Players at Lords. The following year he scored 224 not out for England against Surrey. Other noteworthy batting achievements were over 200 twice in 1871 and 288 for Gloucestershire v. Somerset in 1895. With his brothers E. H. and G. F. he started the Gloucestershire County cricket eleven in 1870. He died in 1915 and the *British Medical Journal* in his obituary claimed that he was admittedly the greatest cricketer whoever played our national game. 'He was not only a great bat, his bowling was a superb sight, all the more so perhaps because it was by no means graceful, but the power he exerted and the skill with which he overcame the batsmen were alike sufficient to win the admiration of the expert and of the ignorant spectator.' His fame was worldwide. In a

**Figure 9**

William Gilbert Grace taverna on Corfu there is a caricature of him at the wicket with the following lines:

Dr. W. G. Grace
Had hair all over his face
Oh! How the crowd cheered
When the ball disappeared
In to his beard.

### JAMES GREIG SMITH

In May 1880 Sir Joseph Lister (later Lord Lister) visited the Bristol Royal Infirmary and gave a demonstration of his 'antiseptic' surgery operating under the carbolic spray. Amongst the audience was a junior surgeon James Greig Smith (Figure 10). He was quick to realise the significance and value of Lister's technique and the possibilities which it opened out for the development of abdominal surgery. Born in Aberdeen in 1854 he entered the local University as an Arts student and graduated M.A. with honours in 1873. Transferring to medicine, he qualified M.B., C.M., with Honourable Distinction in

**Figure 10**

James Greig Smith

1876. That year he came to Bristol as assistant House Surgeon at the Royal Infirmary and served that Institution with devotion for the rest of his life. After various house appointments he was elected Surgeon in 1879. His famous book on Abdominal Surgery was published in 1887 when he was only 33 years old. Five editions of this were called for in the next nine years and it was translated into French. He is described as being very rapid in diagnosis and a forcible, lucid and thorough teacher. Various honours came his way, the Fellowship of the Royal Society of Edinburgh (on the nomination of Lister) in 1883 and of the American Association of Obstetricians and Gynaecologists in 1888. In addition to surgery he was very interested in medical journalism and when the Royal Infirmary published its first and only volume of Reports he acted as surgical editor. He was very largely responsible for the establishment of the *Bristol Medico-Chirurgical Journal* in 1873 and was its first editor. His Presidential Address to the Medico-Chirurgical Society in 1893 was on 'Modern Medical Journalism'. In this he pleaded for a subsidy for a Journal of British Medicine which should be published monthly 'to read which would be enough to keep us abreast of all new knowledge that is true knowledge'. He went on to deplore much that was then published as mere scribbling and written only in self interest. Much of which rings true today. He died aged 43 in 1897 and did not live to use the operating theatre at the Royal Infirmary which he designed but which served as a lasting memorial to him, being still in use nearly 80 years later.

Greig Smith was an enthusiastic, but perhaps not very efficient golfer. With some friends he planned an 18 hole golf course in the vicinity of Woodspring Priory, near Kewstoke. There a private club, limited to eight members, played golf mainly, apparently, at the weekends. Greig Smith wrote a number of essays describing the course, the eight members and a fairly full history of Woodspring Priory. The members were all given 'adoptive' names such as the Epicure, the Prior and the Man of no Possessions. Greig Smith himself was known as the Professor because of his tendency to try to explain a proper golf swing in terms of anatomy. After his death the essays were printed for Private Circulation under the title of 'Woodspring' by J. Greig Smith and published by Arrowsmith in 1898. An introduction by an anonymous author describes Greig Smith as a keen student of nature, always observing some new thing even when playing golf.

**Figure 11**

Edward Long Fox Junior

### EDWARD LONG FOX JUNIOR

The grandson of the founder of Brislington House, Edward Long Fox junior (Figure 11), was born in 1832. After school at Bath Grammar School and then Shrewsbury he went to Balliol College, Oxford, where he obtained a first class in the Natural Science Tripos. He then studied medicine at Edinburgh and at St. George's Hospital, graduating M.B. in 1857. The same year he was appointed physician to the Bristol Royal Infirmary. It is recorded that on his first ward round he said to the students: 'I wish to say that as I have only just passed out of the student stage myself, I shall feel greatly pleased should any of you notice anything overlooked in my walk and practice here that might be of importance in treating patients, if you would kindly remind me of the fact . . .' He obtained his M.D. in 1861 and was elected F.R.C.P. in 1870. When Clifton College was founded in 1862 he became the first physician. His Presidential Address to the Bristol Medico-Chirurgical Society in 1881 was on the Medulla Oblongata. In 1882 he gave the Bradshaw Lecture at the Royal College of Physicians on the 'Influence of the Sympathetic System in Disease'. This was later expanded into a book 'Influence of the Sympathetic in Disease' published in 1885. In a paper in the Bristol Medico-Chirurgical Journal in 1884 on 'The Nature and Treatment of Chorea' he recognised the association of chorea and acute rheumatism and noted the changes in the mitral valve seen on post-mortem in cases of chorea. This led him to speculate, incorrectly in fact, that chorea might be caused by emboli from the mitral valve lodging in the brain. President of the British Medical Association when it held its annual meeting in Bristol in 1894, his presidential address dealt with Medicine and the State. He was always a persistent advocate of abstinence from alcohol and was for some years President of the National Temperance League. Nevertheless he was popular with the students perhaps because he invited them all to a strawberry tea in his house in Clifton every summer. Under the then rules he retired from the Infirmary staff after 20 years in 1877, but continued active until his death in 1902. His many friends and admirers subscribed to found an annual lecture in his memory, the first of which was given, as has already been mentioned, in 1904 by Dr. John Beddoe on 'The Ideal Physician.'

### FRANCIS RICHARDSON CROSS

By 1880 the Bristol Eye Hospital, which had been founded seventy years before largely due to the enthusiasm and drive of William Henry Goldwyer, had sunk to a very low ebb both financially and professionally. But in 1882 it received a great stimulus and was virtually rejuvenated by the appointment of F. Richardson Cross (Figure 12) to the staff as surgeon. When he retired in 1925 after 43 years devoted service to the hospital he had made it one of the leading Eye Hospitals in the country.

**Figure 12**

Francis Richardson Cross

Unhappily he did not live to see the new building which he had done so much to create as it was not completed until four years after his death. He was born at Merriott in Somerset where his father was vicar and went to Crewkerne Grammar School. As a student at King's College Hospital, London, he won the 100 yards race in the interhospital sports in a record time and held this amateur record for a year. After qualifying he paid post-graduate visits to Vienna, Berlin, Paris and Utrecht. He was appointed assistant surgeon at the Royal Infirmary in 1878, but resigned from this in 1885 when the Infirmary created a department of Ophthalmology and he was appointed Ophthalmic Surgeon. After 15 years he retired from the Infirmary to devote all his time to the Eye Hospital. He was Dean of the Medical School of University College, Bristol, for 13 years and a member of the Council of the Royal College of Surgeons from 1898 to 1914. President of the Bristol Medico-Chirurgical Society in 1891, his Presidential Address on Progress in Medicine stressed the need for special departments to be developed in all large hospitals. In 1912 he was awarded the honorary degree of Doctor of Laws by the University of Bristol. He was not interested in the eye alone, but in all aspects of the phenomenon of sight. In his Long Fox lecture on the Evolution of 'The Sense of Sight' he traced the development of an organ of sight from the lowest forms of life to man and in 1909 his Bradshaw Lecture at the Royal College of Surgeons dealt with the brain structures concerned with vision and the visual field. In addition to his ophthalmological work, which was recognised by his election as President of the Ophthalmological Society of the United Kingdom in 1914, he took an interest in civic affairs and was Sheriff of Bristol in 1898.

### PATRICK WATSON WILLIAMS

Patrick Watson Williams (Figure 13) was one of the leaders of that small band of pioneers who changed the study of the ear, nose and throat from a casual occupation of the general physician or surgeon into a highly developed speciality. Born in Clifton in 1863 where his father was in practice, he was educated at Clifton College and entering the Medical School of University College, Bristol, he qualified in 1884. It is curious how sometimes apparently minor events play a large part in determining a man's subsequent career. After various resident appointments Watson

**Figure 13**

Patrick Watson Williams

Williams applied for the post of Honorary Obstetrician to the Bristol Royal Infirmary. The appointment was hotly contested and Watson Williams unhappy at and disapproving of some of the methods used by those opposing his election withdrew his application. Instead in 1888, a year later, he was appointed assistant physician and became a full physician 17 years later. However, while fulfilling his duties as a physician be became more and more interested in diseases of the nose and throat. In 1894 his book on 'Diseases of the Upper Respiratory Tract' appeared and ran into several editions. In 1906 the Royal Infirmary established a Department of rhinolaryngology and Watson Williams resigning his post as physician was appointed the first surgeon in charge of the new Department. For some years the general surgeons refused to relinquish their interest in otology, but finally they relented and the Department became one of oto-rhino-laryngology. Watson Williams continued as the senior member of this until his retirement in 1921. He became a firm believer in the importance of 'focal sepsis' in the causation of many conditions. His views on this were set out in his book 'Chronic Nasal Sinusitis and its Relation to General Medicine' published in 1930. This was an expansion of the Semon Lecture which he gave in 1925. Various honours came his way and in 1927 he was Président d'Honneur de la Societé francaises d'Otologie et Laryngologie at its meeting in Paris, and in 1934 the University of Bristol awarded him the honorary degree of Doctor of Medicine. He did much for the Bristol Medico-Chirurgical Journal as assistant editor from 1900 to 1912 and editor from 1912 to 1926. It was only his unbounded enthusiasm which kept the journal alive during the 1914–1918 war. President of the Medico-Chirurgical Society in 1913 he gave his Presidential Address on 'Specialisation in the Medical Curriculum'. He was a splendid host and was noted for giving 'scrumptious banquets' which he always seemed to enjoy as much as his guests. He died in 1938.

### CAREY FRANKLIN COOMBS

Another West Countryman to gain distinction in medicine in Bristol was Carey Franklin Coombs (Figure 14), who was born at Castle Cary in Somerset, where his father was in practice, in 1879. After attending Keyford School, Frome, he entered the Bristol Medical School in 1896, later transferring to St. Mary's Hospital, London, he graduated M.B., B.S., in 1901 and proceeded M.D. in 1903. Moving to Bristol, he was appointed Assistant Physician to the General Hospital in 1907. He became a Member of the Royal College of Physicians in 1908 and was elected to the Fellowship in 1917. At St. Mary's he

**Figure 14**

Carey Franklin Coombs had been greatly influenced by F. J. Poynton and his research on Acute Rheumatism and determined to continue this work in Bristol. During the 1914–18 war he served with the R.A.M.C. in Mesopotamia and France. His great book, on Rheumatic Heart Disease, which is now a classic, was published in 1924 and dealt exhaustively with the clinical features and pathology of that condition. Following this in 1927 he obtained grants from the Harmsworth Trust and from the Colston Research Society to establish the University Centre of Cardiac Research at the General Hospital. The Hospital assisted by making accommodation available in the octagon above the Board Room. This had previously been divided into cubicles to provide bedrooms for nurses. These cubicles were converted into a small histology laboratory, three study/offices and an electrocardiograph room. The adjoining bathroom, with a board over the bath, served as the dark room necessary for the processing of the cardiograms which at that time were recorded on a glass photographic plate and then had to be printed on paper. The University, through the Department of Physiology, provided the electrocardiograph. Here he directed further work on acute rheumatism, on bacterial endocarditis and cardiovascular syphilis. He was one of the first in the West of England to make a diagnosis of coronary thrombosis which was confirmed by a post-mortem carried out in the patient's house. In all he did, he did with zest and enthusiasm and it may be claimed that he lived every moment of his life doing nothing to spare himself. In an effort to improve the case of the child suffering from acute rheumatism he persuaded the Crippled Children's Society that such a child was equally crippled as one who had a paralysed leg and threw himself wholeheartedly in promoting the foundation of the Orthopaedic Hospital at Winford, where at one time three quarters of the patients were suffering from acute rheumatism and rheumatic heart disease. Although his main interest was Rheumatic heart disease, he was concerned with all aspects of cardiology. In 1926 in his Long Fox Lecture he dealt with the 'Aetiology of Cardiac Disease' and the following year he gave the Chadwick Lecture on 'Cardiac Disease and its relation to Industrial Efficiency'. Another of his crusades was the amalgamation of the Bristol Royal Infirmary and the Bristol General Hospital and he was one of the chief architects of this fusion, but unhappily he died eight years before it was effected. In 1930 he was Lumleian Lecturer at the Royal College of Physicians and in these lectures dealt exhaustively with all aspects of Cardiovascular Syphilis. The following year he was elected to the Council of the College, but died shortly afterwards at the tragically early age of 52. It is interesting to reflect that 40 years after his death the two diseases to which he gave so much study – acute rheumatism and cardiovascular syphilis – were practically extinct in Great Britain. After his death friends and colleagues subscribed to found a memorial lecture to be given in the University every two years. The first of these was given by Mr. Laurence O'Shaughnessy in 1937 on the 'Pathology and Surgical Treatment of Cardiac Ischaemia' in which he described the operation of cardio-omentopexy.

### ERNEST HEY GROVES

Ernest William Hey Groves (Figure 15) was born in India in 1872, the son of a civil engineer. He was educated at Redland Hill House School in Bristol. Then winning an entrance scholarship to St. Bartholomew's Hospital Medical school he graduated B.Sc. in 1890 at the age of 18! He then demonstrated biology for three years before proceeding to qualify M.R.C.S., LR.C.P., in 1895, M.B., B.S., in 1897, and M.D. in 1900. Coming to Bristol, he settled in practice in Kingswood where with his wife, who had been a sister at Barts, as nurse and matron he ran a small nursing home in his own house. Much to some people's surprise he was

**Figure 15**

Ernest Hey Groves invited to become assistant surgeon to the General Hospital in 1903, without the Fellowship of the Royal College of Surgeons. However, two years later he attained this, together with the M.S. with the Gold Medal. From the start, possibly influenced by the work of Sir Robert Jones, he was interested in the surgery of bones and joints, and even while at Kingswood had been anxious to obtain dogs and cats with fractures in order to study bone healing. He was one of the first to realise that complete immobilisation of a fracture might result in delayed union and rapidly became a pioneer in all types of bone surgery and grafting. However he continued to carry out his full duties as a general surgeon. He served in the R.A.M.C. in the 1914–18 war and while in Egypt he not only designed a special splint for fractured femurs, which must have saved many lives, but also organised its manufacture by Egyptians in Alexandria. At the Royal College of Surgeons he was a member of Council for 23 years and Vice-President from 1928 to 1929. He gave the Bradshaw Lecture on 'Reconstructive Surgery of the Hip' in 1926 and the Hunterian Oration in 1930 on 'Hero Worship in Surgery'. Like Carey Coombs he did much to further the amalgamation of the Infirmary and Hospital and when appointed Professor of Surgery in 1922 he insisted on doing ward rounds for the students at both hospitals on alternate weeks contrary to all custom and tradition. Perhaps his greatest achievement was the creation of the *British Journal of Surgery*. He played a large part in its foundation in 1913 and for 20 years was its indefatigable editorial secretary. Many honours came his way. He was President of the British Orpthopaedic Association in 1928 and of the Association of Surgeons of Great Britain and Ireland in 1929. President of the Bristol Medico-Chirurgical Society in 1932 his Presidential Address was entitled 'A Surgical Adventure (an autobiographical sketch)'. On the occasion of the Centenary of the Bristol Medical School he was awarded the honorary degree of Doctor of Science by the University of Bristol. He also received honorary degrees from Queen's University, Belfast, and from the National University of Ireland. Hey Groves was a very generous person, although his generosity was usually concealed and more than one impecunious medical student was enabled to qualify by his financial assistance. A great individualist he had no use for red tape. It is recorded that when going on board the ship that was to take him to Alexandria during the 1914–18 war he was told that no R.A.M.C. Officer could embark unless properly dressed and wearing spurs. He scoured the docks and found a rusty pair of spurs in a marine store, put them on and went on board. He then tossed them ashore repeatedly for the use of his colleagues, each of whom used them in turn. He loved travel and was a founder member of the Moynihan Travelling Surgical Club. He pursued 'play' with the same zest that he put into his work. Not only was he an enthusiastic golfer – to encourage this he instituted a golf competition for the staff of the two hospitals – but he could often be seen doing the skater's waltz at the local ice rink or swimming in the pool at Clevedon. He died in 1944 after a long illness.

It is thus clear that over two and a half centuries Bristol can claim to have had a succession of doctors distinguished in many different fields, whose contributions are still remembered today. Many of them came from the West Country and many were born into medical families.

### REFERENCES

BUTCHER, E. E. The Bristol Corporation of the Poor, Bristol Records Society, Vol. 3, has details of St. Peter's Hospital.

DEWHURST, K. The Quicksilver Doctor, Bristol, 1957, and Thomas Dover's Life and Legacy, New York Academy of Medicine, deals fully with Thomas Dover.

EYLES, V. A. Scientific activity in the Bristol Region in the past. In MacInnes, C. M. and Whittard, W. F., Bristol and its Adjoining Counties, British Association for the Advancement of Science, deals fairly fully with Thomas Beddoes and the Pneumatic Institute.

LITTLE, B. The City and County of Bristol, London, 1954, has references to Thomas Dover, E. Long Fox senior, Thomas Beddoes, J. Addington Symonds.

MUNRO Smith, G. The History of the Bristol Royal Infirmary, Bristol, 1917. Contains much information about John Bonython, E. Long Fox senior and E. Long Fox junior, Thomas Beddoes, J. C. Prichard, William Budd, John Beddoe, J. Greig Smith, F. Richardson Cross and P. Watson Williams.

NIXON, J. A. *Bristol Med.-Chi.Journ.* 1909, 27, 31 and *B.M.J.* 1913.1.619 supplements this.

SYMONDS, J. A. Some Account of the Life, Writings and Character of the late James Cowles Prichard is a full biography. This is supplemented by personal recollections by Alford, H., *Bristol Med.-Chi.Journ.* 1890, VIII.

SYMONDS, J. A. junior, Miscellanies is a full record of J. A. Symonds senior.

The Dictionary of National Biography has records of Thomas Dover (Vol. XV), Thomas Beddoes (Vol. IV), J. C. Prichard (Vol. XLVI), J. Addington Symonds (Vol. LV), John Beddoe (Vol. 1901–11), W. G. Grace (Vol. 1912–21).

Goodall, E. W. William Budd, Bristol, 1936, Beddoe, J. Memories of 80 years (Autobiography) 1910, Keith, Sir A. *Bristol Med.-Chi.Journ.* 1930, XLVII, 287 (Beddoe Memorial Lecture), have all been consulted, together with the following obituary notices:

J. A. Symonds, *B.M.J.* 1871, vol. II; William Budd, *B.M.J.* 1880, vol. I; W. G. Grace, *B.M.J.* 1915, II, 661; and *Bristol Med.-Chi.Journ.* 1915, vol. XXXII, 255; J. Greig Smith, *Bristol Med.-Chi.Journ.* 1897, Vol. XV, 105; E. Long Fox junior, *Bristol Med.-Chi.Journ.* 1902, vol. XX, 97; F. Richardson Cross, *Bristol Med.-Chi.Journ.* 1913, 48, 226; Carey F. Coombs, *B.M.J.* 1932, vol. II, 1126 and 1171, *Bristol Med.-Chi.Journ.* 1932, 49, 326; E. W. Hey Groves, *Lancet* 1944, vol. II, 613, *Bristol Med.-Chi.Journ.* 1945, 62, 31, and for the last three Who was Who 1929–40 and 1941–50.

## Figures and Tables

**Figure 1 f1:**
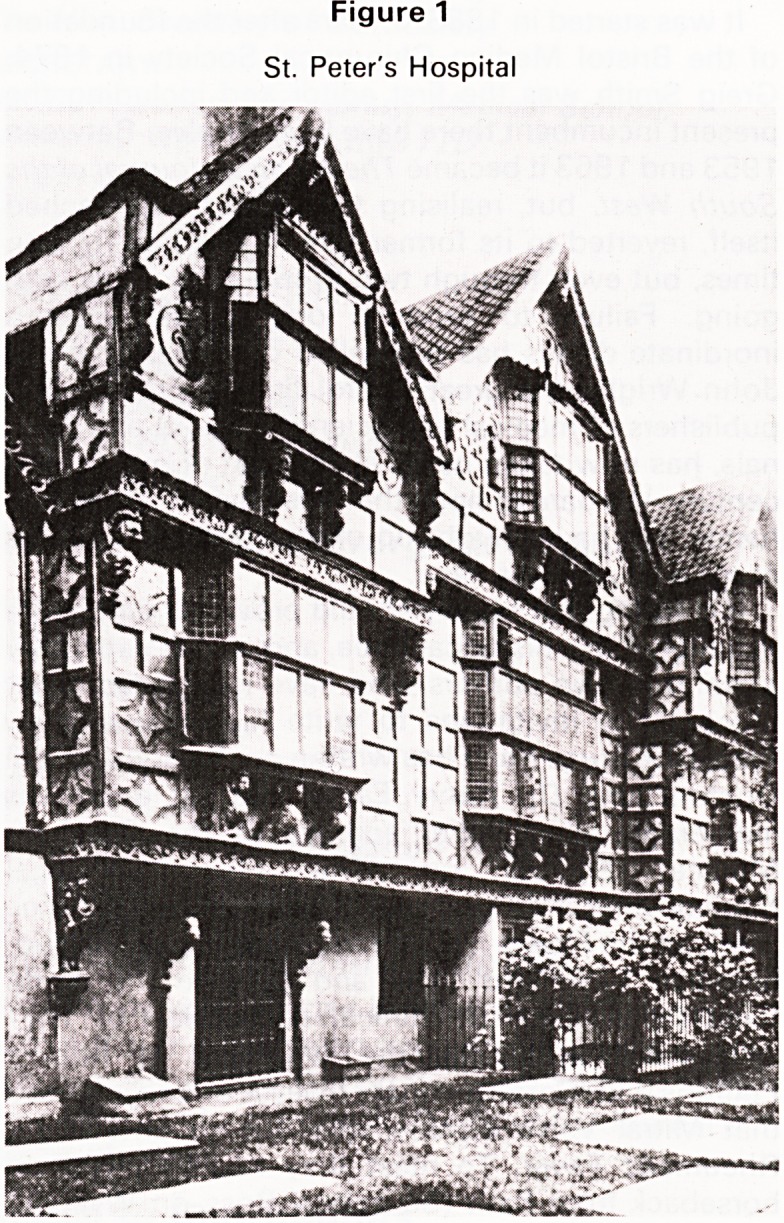


**Figure 2 f2:**
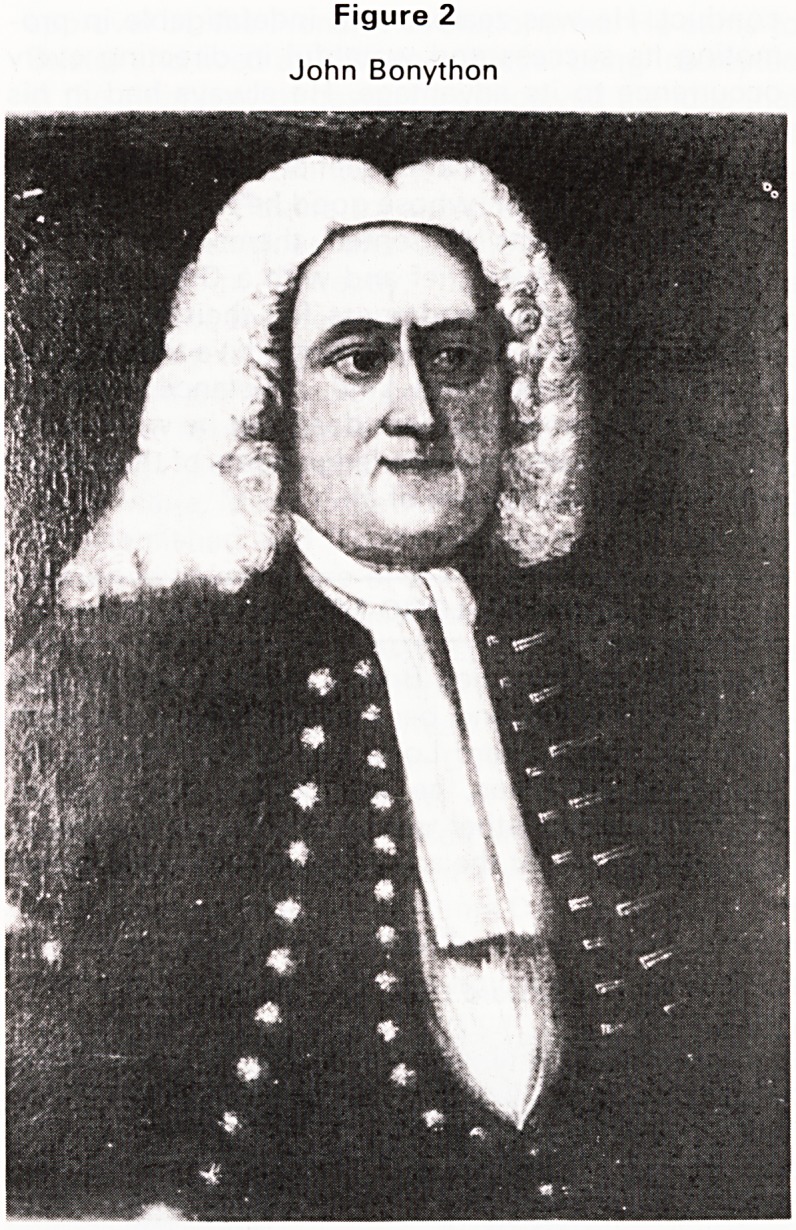


**Figure 3 f3:**
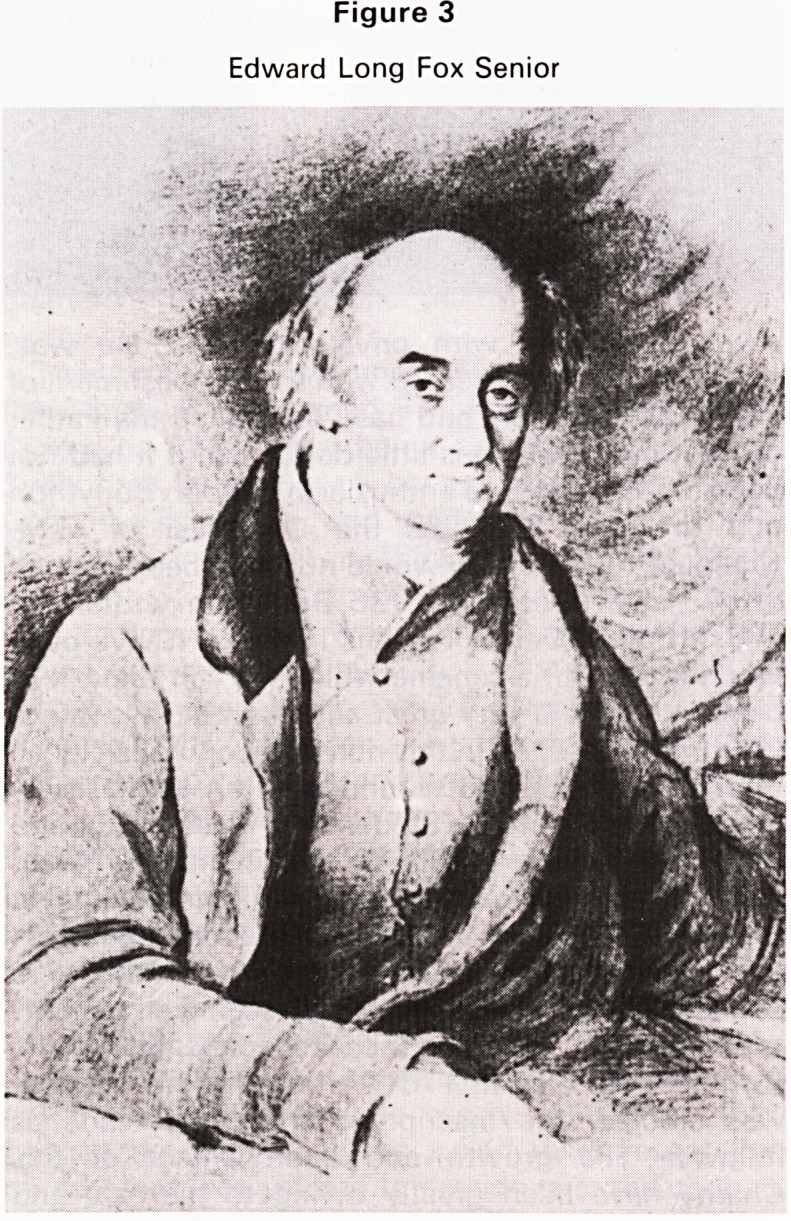


**Figure 4 f4:**
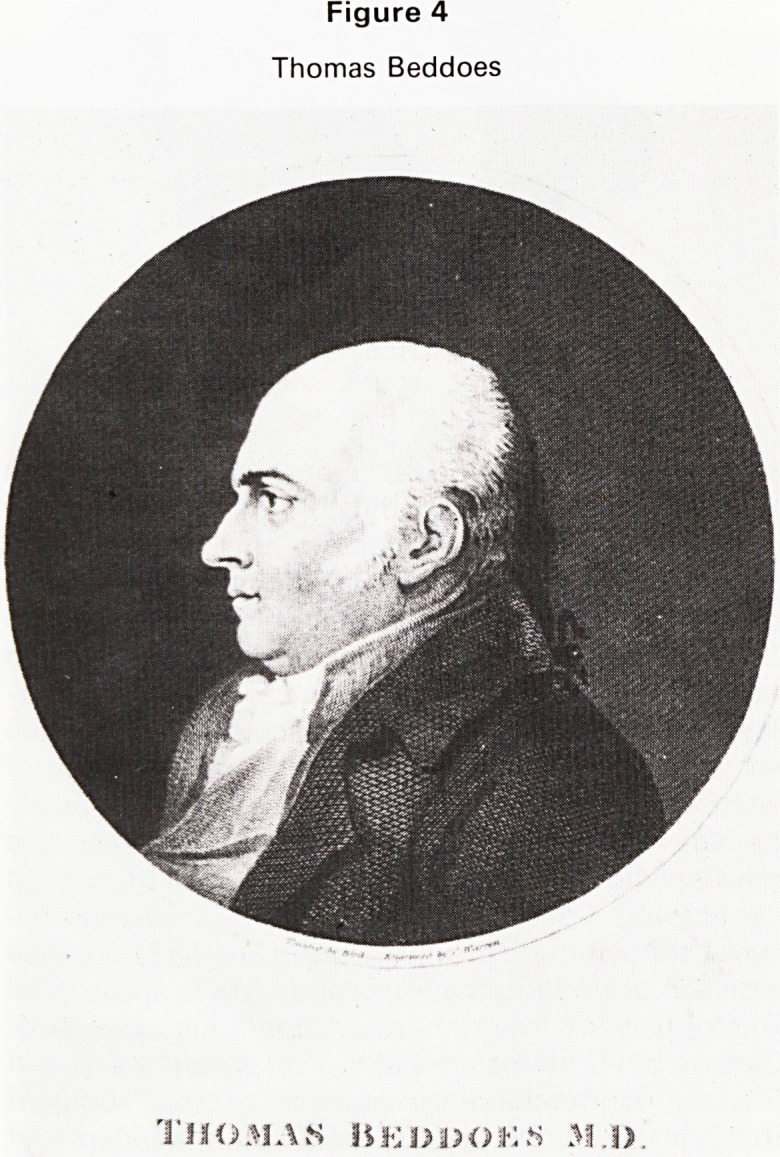


**Figure 5 f5:**
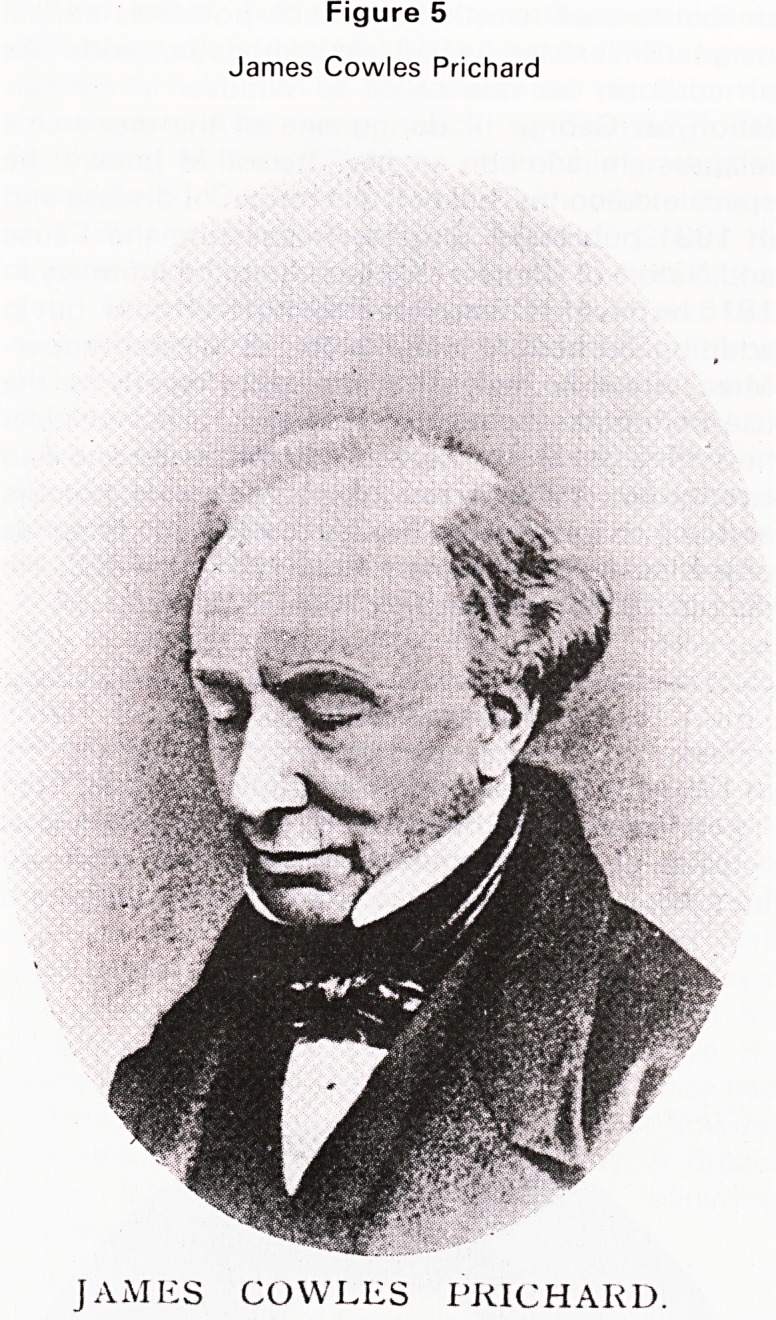


**Figure 6 f6:**
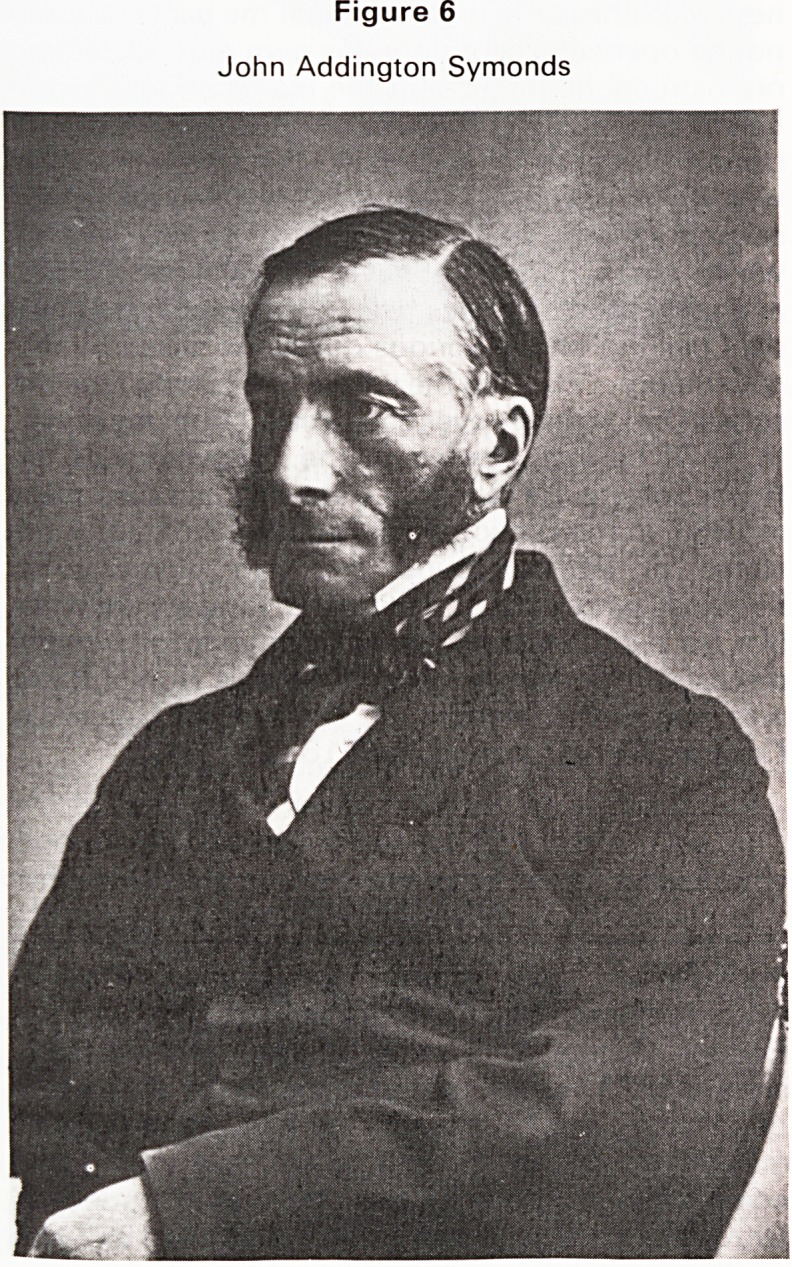


**Figure 7 f7:**
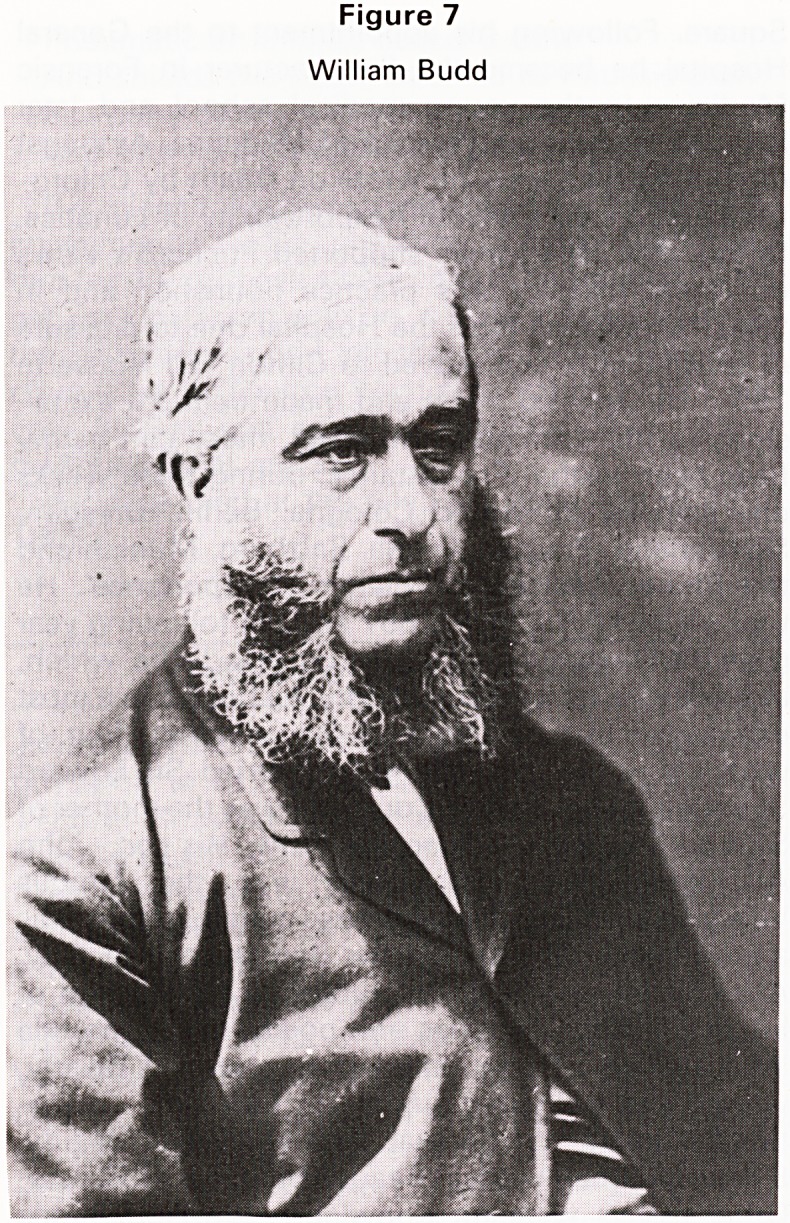


**Figure 8 f8:**
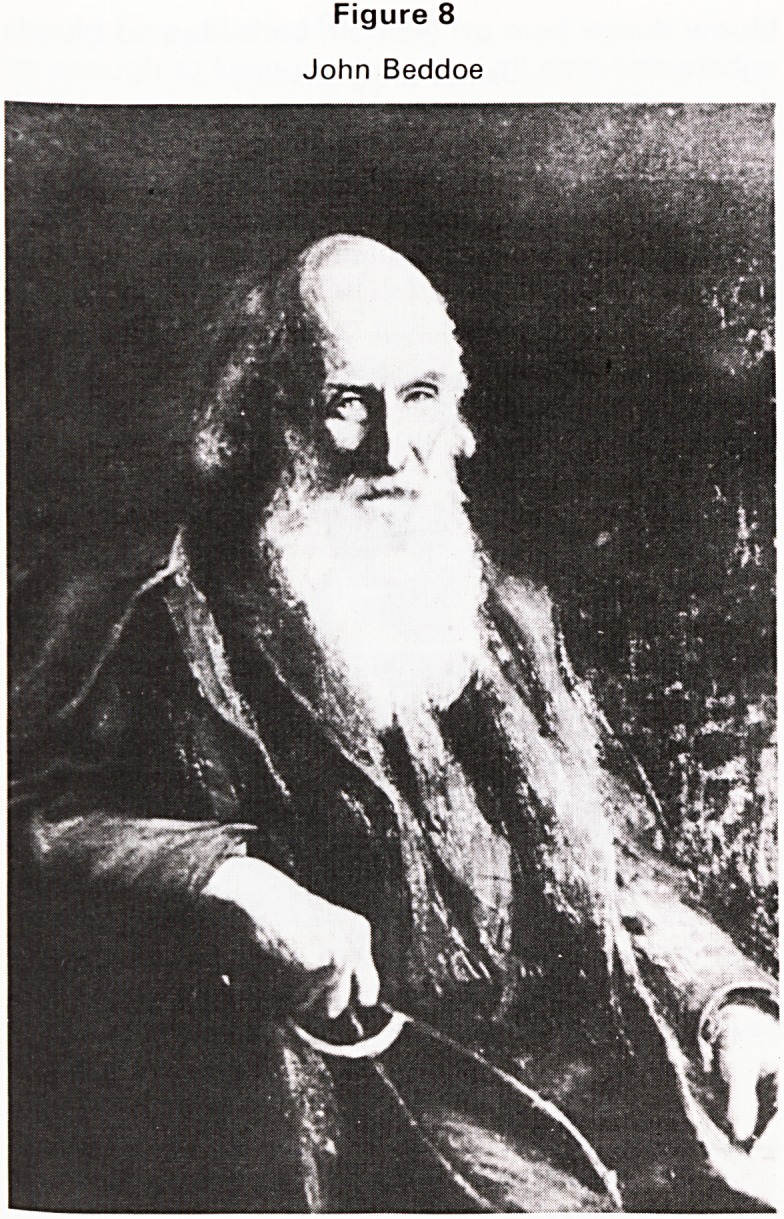


**Figure 9 f9:**
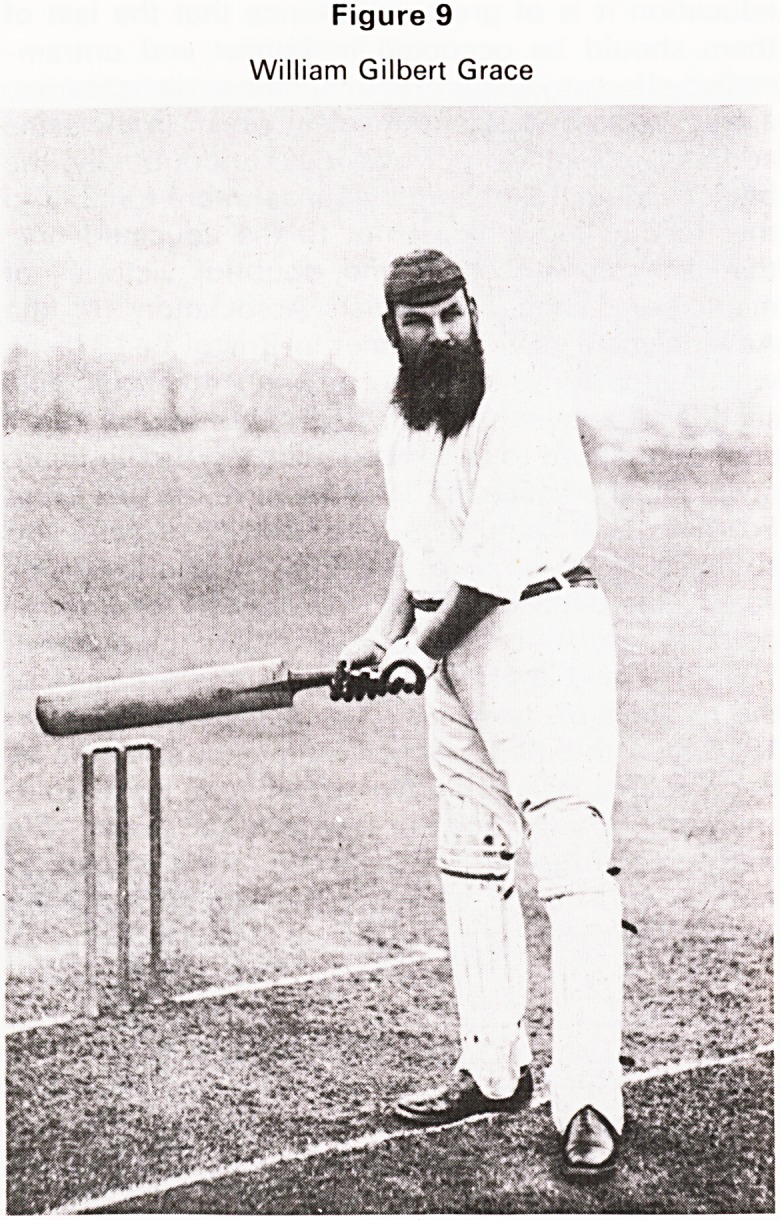


**Figure 10 f10:**
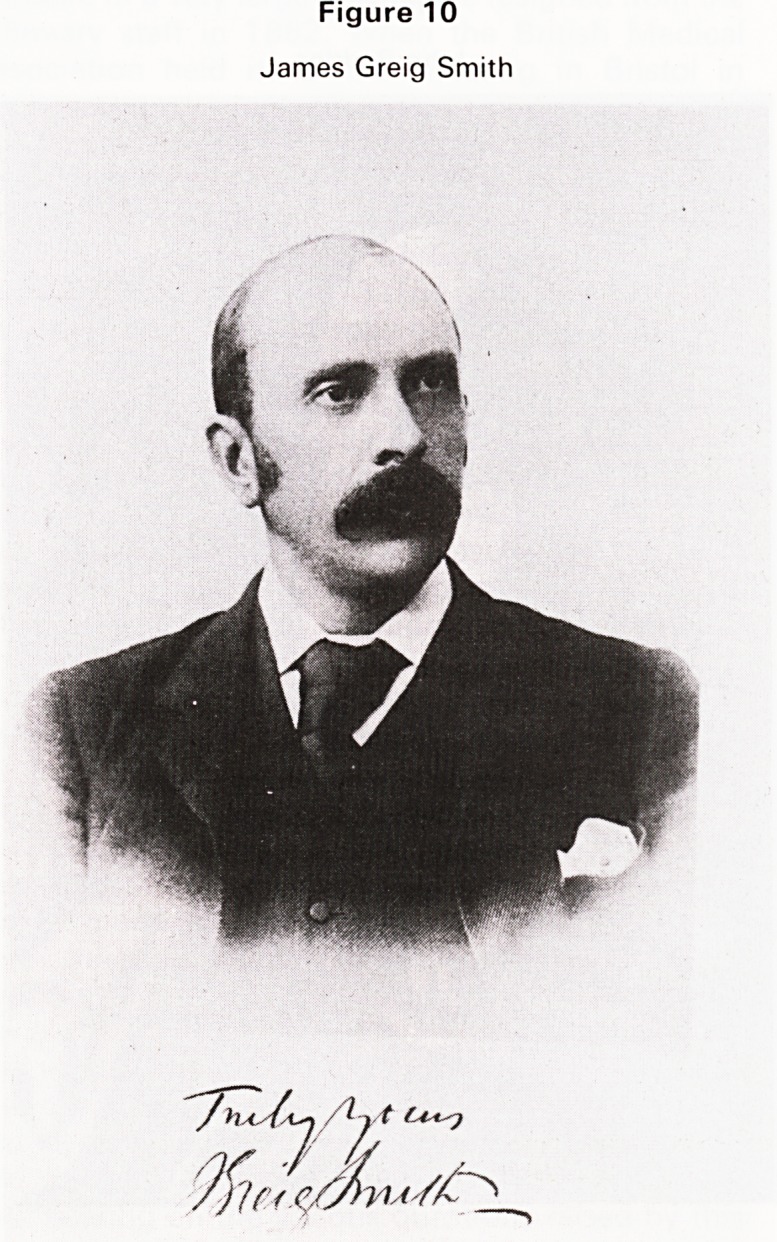


**Figure 11 f11:**
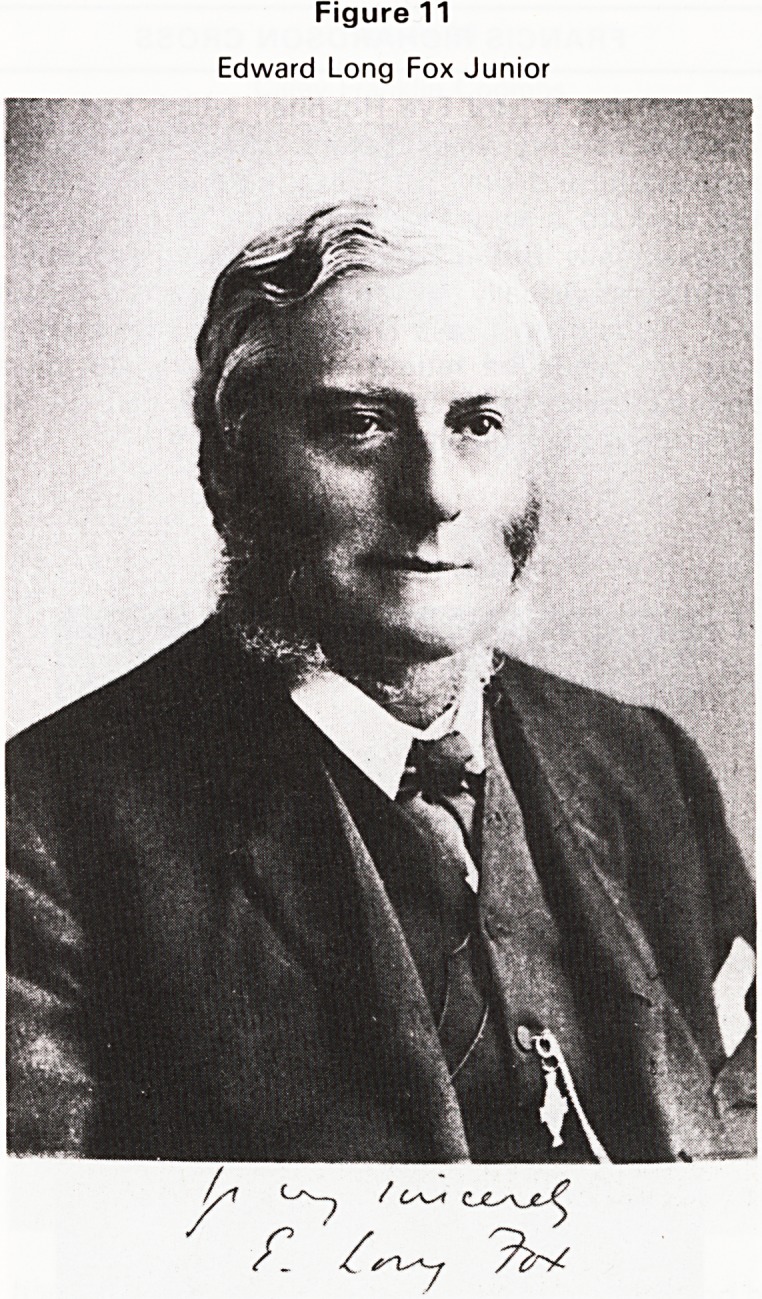


**Figure 12 f12:**
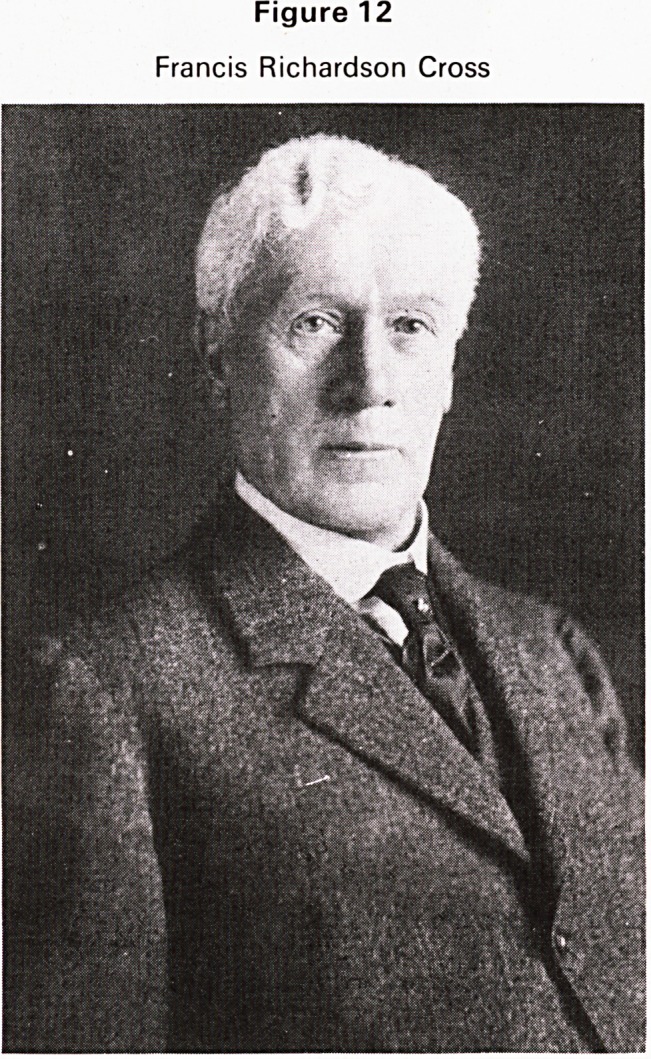


**Figure 13 f13:**
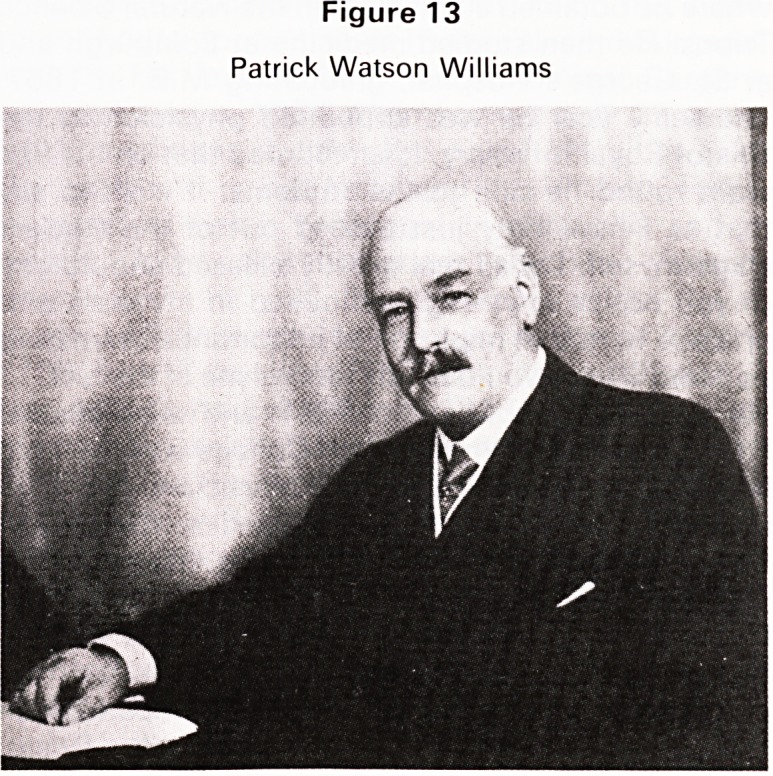


**Figure 14 f14:**
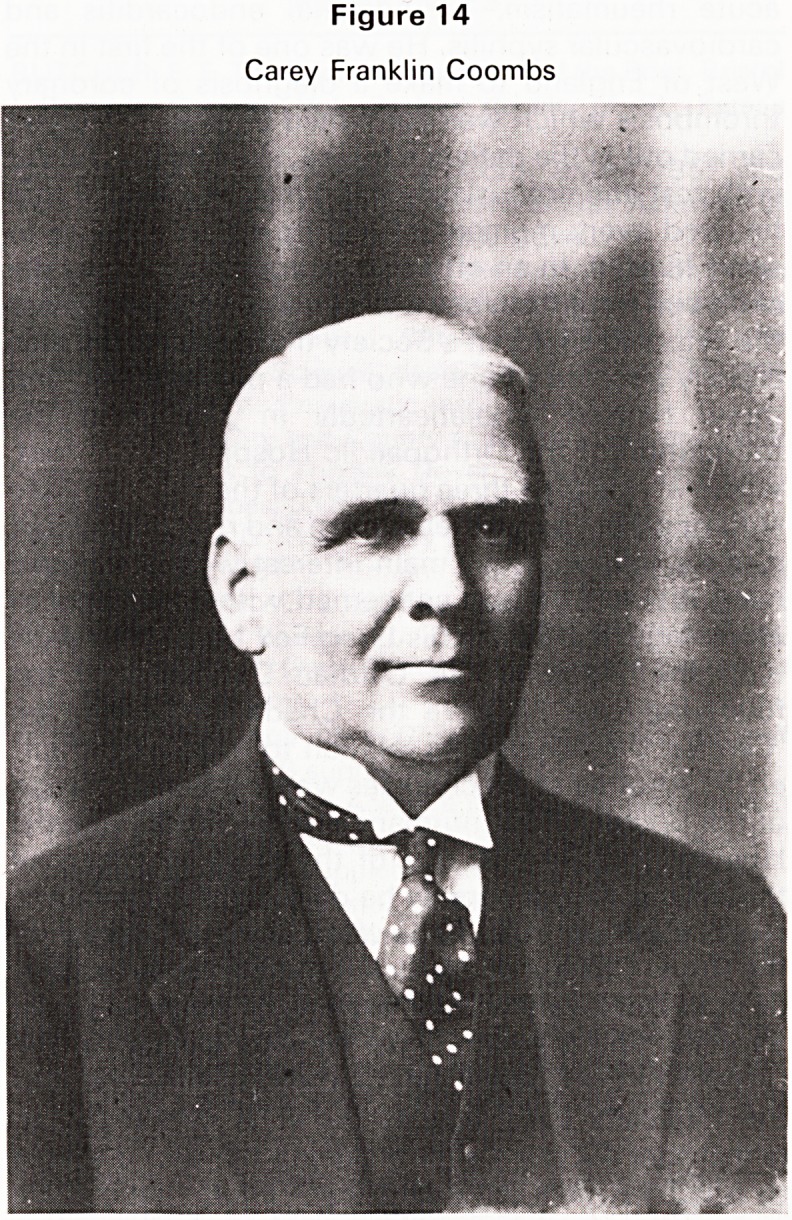


**Figure 15 f15:**